# Characteristics of Sucrose Transport through the Sucrose-Specific Porin ScrY Studied by Molecular Dynamics Simulations

**DOI:** 10.3389/fbioe.2016.00009

**Published:** 2016-02-15

**Authors:** Liping Sun, Franziska Bertelshofer, Günther Greiner, Rainer A. Böckmann

**Affiliations:** ^1^Computational Biology, Department of Biology, Friedrich-Alexander University of Erlangen-Nürnberg, Erlangen, Germany; ^2^Computer Graphics Group, Department of Computer Science, Friedrich-Alexander University of Erlangen-Nürnberg, Erlangen, Germany

**Keywords:** molecular dynamics, ScrY, porin, sucrose binding, transport mechanism, potential of mean force

## Abstract

Sucrose-specific porin (ScrY) is a transmembrane protein that allows for the uptake of sucrose under growth-limiting conditions. The crystal structure of ScrY was resolved before by X-ray crystallography, both in its uncomplexed form and with bound sucrose. However, little is known about the molecular characteristics of the transport mechanism of ScrY. To date, there has not yet been any clear demonstration for sucrose transport through the ScrY. Here, the dynamics of the ScrY trimer embedded in a phospholipid bilayer as well as the characteristics of sucrose translocation were investigated by means of atomistic molecular dynamics (MD) simulations. The potential of mean force (PMF) for sucrose translocation through the pore showed two main energy barriers within the constriction region of ScrY. Energy decomposition allowed to pinpoint three aspartic acids as key residues opposing the passage of sucrose, all located within the L3 loop. Mutation of two aspartic acids to uncharged residues resulted in an accordingly modified electrostatics and decreased PMF barrier. The chosen methodology and results will aid in the design of porins with modified transport specificities.

## 1 Introduction

Most bacteria produce cell walls surrounding the cytoplasmic membrane to protect their vulnerable cell structure and to maintain their mechanical rigidity. Gram-negative bacteria possess an outer membrane surrounding the inner cell wall with a peptidoglycan layer in between (Glauert and Thornley, 1968). The outer membrane acts as a selective permeability barrier to exclude noxious compounds and exchange nutrients and waste products with the external medium (Nikaido and Nakae, 1980). For this purpose, the outer membrane contains porins, a special class of proteins, which function as channels across the membrane. Through porins a variety of substrates can pass in a diffusion-like process (Nakae, 1976). Such porins can be classified into (i) general diffusion porins that are responsible for the non-specific and spontaneous transport of ions and small hydrophilic molecules, and (ii) specific diffusion channels that contain stereospecific binding sites within the pore, facilitating the uptake of solutes of certain types (Nikaido, 1992). The expression of these specific porins is usually induced under special environmental conditions (Nikaido and Vaara, 1985).

General diffusion porins usually form tightly assembled homotrimers. Each subunit is a water-filled *β*-barrel consisting of typically 16 or 18 antiparallel *β*-strands oriented perpendicular to the membrane plane and tilted by 30–60° with respect to the molecular symmetry axis (Nabedryk et al., 1988; Jap, 1989). The *β*-strands are connected by short turns on the periplasmic side and by long loops on the external side (Nikaido, 1994). Interestingly, porin channels are constricted by the so-called eyelet loop that folds inwardly and is attached to the inner side of the barrel wall, resulting in a cross-section of a minimal size of ~7 × 11 Å, which allows the passage of hydrophilic solutes up to an exclusion size of ≈600 Da (Weiss et al., 1991; Schirmer, 1998). Since the pore size is similar to the diameter of most nutrient molecules, the diffusion rates are strongly affected by the physical properties of the substrates. As typical general porins in *E. coli*, OmpF and OmpC were reported to favor both neutral molecules and cations, while PhoE favors anions (Nikaido and Vaara, 1985; Bauer et al., 1989).

One example of a specific porin is the sucrose-specific porin (ScrY) of enteric bacteria, expressed when *E. coli* is starved for sucrose (Schmid et al., 1982). It permits the rapid influx of sucrose across the outer membrane, allowing cells to grow on sucrose as a solo carbon source (García, 1985; Schmid et al., 1991). ScrY was found during the investigation of the plasmid-encoded metabolic pathway of sucrose in *Salmonella typhimurium*, where sucrose has only a small rate of translocation through the outer membrane in the absence of the *scr* genes pUR400 (Schmid et al., 1988). The crystal structure of sucrose-specific porin was determined at a resolution of 2.4 Å, both in its uncomplexed form and with bound sucrose (Forst et al., 1998). Apart from the shared architectural properties with general porins, each polypeptide chain of the ScrY channel, containing 413 structurally well-defined amino acids, traverses the membrane 18 times as antiparallel *β*-strands surrounding a hydrophilic pore. Importantly, the eyelet loop (L3) folds inwardly into the lumen of the *β*-barrel forming a selective gate, comparable to general porins as mentioned above. This is in excellent agreement with the physiological function of the porin to exclude toxic compounds and to maximize the uptake of nutrients using wide openings and a greasy pathway for sugars (see below), minimizing frictional interactions to the contriction site in the L3 region (Welte et al., 1995). Sucrose-specific porin binds two sucrose molecules at the same time in a certain configuration (Forst et al., 1998) [similar to maltoporin (Dutzler et al., 1996)]. Besides, it also has the features of a general diffusion pore with a comparable single-channel conductance, which is much smaller for other specific porins (Schmid et al., 1991; Schülein et al., 1995).

The essential role of the inner loop L3 for modulating the translocation of molecules was also suggested for the outer membrane porin OprD, specific for the uptake of small natural substrates like cationic amino acids, by means of *in silico* electrophysiology and metadynamics simulation techniques (Samanta et al., 2015). A combination of *in silico* and *in vitro* studies was used in the study of the outer membrane channels OprP (specific for phosphate transportation) and OprO (specific for diphosphate transportation). Two amino acids in the central constriction region were suggested to generate the substrate specificity. Reciprocal exchange of these amino acids resulted in an interchange of substrate specificities for these channels (Modi et al., 2015). For OmpF, the cation selectivity was reported to be highly influenced by the electrostatic environment of the constriction region. It was found that removing the cationic residues in the cross-sectional area enhanced the cation selectiviy, whereas removal of the anionic residues reversed the selectivity (Pezeshki et al., 2009).

Another specific porin, Maltoporin (LamB), was first identified as the receptor for *λ*-phage in *E. coli* (Randall-Hazelbauer and Schwartz, 1973). It forms trimeric channels, which are specific for the transport of maltose and malto-oligosaccharides and is synthesized for maltodextrin concentrations below 10 μM (Nikaido and Vaara, 1985; Saurin et al., 1989; Death et al., 1993). Noteworthy, ScrY can also function as a malto-oligosaccharide porin, and LamB is also able to activate the influx of sucrose, but only at high substrate concentrations (Szmelcman and Hofnung, 1975; Schülein and Benz, 1990; Schmid et al., 1991). Therefore, it was suggested that ScrY is a sugar-specific porin with a similar function as LamB. This is further stressed by their structural similarity (Dali Server yields a *Z* score of 36.8) and their sequence similarity (Blast *E* value = 4e − 173) (Altschul et al., 1990; Holm and Rosenström, 2010). Although the similarity in topology is remarkable, the depicted porins share only 23% amino acid sequence homology (Protein Data Bank). The sequence identity is increased at the prospective glucose-binding regions, located in the first half of the primary sequence from the N-terminal end (Hardesty et al., 1991; Schülein et al., 1995). Interestingly, the binding constant of ScrY and LamB with malto-oligosaccharides increases with the number of glucose residues but becomes saturated after five residues, as inferred from current noise and ion flow inhibition studies respectively (Benz et al., 1987; Schülein et al., 1991). Based on these observations, Benz and colleagues proposed that the binding site has a length of about five glucose residues along the wall of the channel and the dextrine penetrates in a single file because of the hourglass-shaped constriction of the pore. However, evidence for this mechanism is still scarce (Benz et al., 1987; Benz and Bauer, 1988).

Very few experiments have been carried out so far that provide insights into the structure–function relationship of ScrY. The first clear demonstration of sucrose uptake was demonstrated by Schmid et al. (1982) in their *in vivo* research of *E coli* using chromatography techniques, where the apparent *K*_m_ value (i.e., the Michaelis constant) was determined to 10 μM. Simultaneously, the binding of sucrose to ScrY has been investigated by Schülein who reported a stability constant K defined as the ratio between on and off rate constants for sugar binding of 20 l mol^−1^ based on the relative rates of permeation of ScrY and LamB and the stability constant of sucrose binding to LamB (Schülein et al., 1995). The penetration rates for sugar through porin channels is likely to be strongly affected by its concentration on each side of the membrane, as revealed by current noise studies of Jordy on LamB due to the blockage of the ionic flow during the passage (Jordy et al., 1996). Besides, the conception of a “greasy slide” was described both for ScrY and LamB containing five or six contiguous aromatic side chains lining up on one side of the channel and forming a smooth hydrophobic path (Schirmer et al., 1995; Wang et al., 1997; Forst et al., 1998). The greasy slide extends from the channel vestibule through the constriction zone to the periplasmic exit and functions as a guidance for sugar molecules sliding through the channel by engagement in non-specific hydrophobic interactions with the pyranose rings (Schirmer et al., 1995). Based on this concept, the passage of sucrose has been hypothesized to consist of a series of steps: first, sucrose diffuses from the external solution to a trapping zone in sucrose-specific porin. Second, a sucrose molecule slides along the greasy slide and enters the binding region. Finally, sucrose passes through the binding region and enters the cell (Forst et al., 1998). However, to date there is no convincing evidence supporting this hypothesis.

In this study, we have investigated the dynamics of the ScrY trimer embedded in a phospholipid bilayer as well as the characteristics of sucrose translocation by means of atomistic molecular dynamics (MD) simulations. Various structural properties of ScrY and its implications for sucrose transport are discussed. Based on results of potential of mean force (PMF) calculations for sucrose transport through ScrY, key residues were pinpointed and a mutant suggested that showed a significantly decreased free energy barrier for the passage of sucrose.

## 2 Computational Methods

### 2.1 System Setup

**Figure 1:**
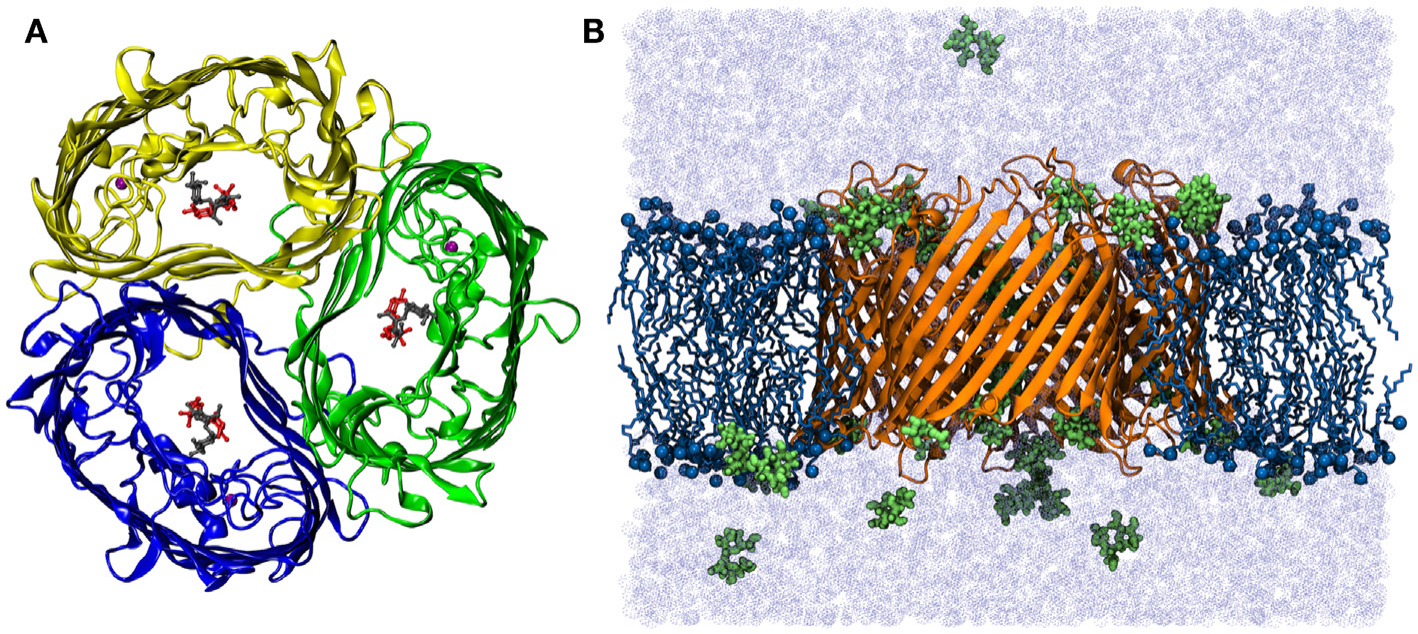
**(A)** Top view of the crystal structure of ScrY (Forst et al., 1998). The monomers of the homo-timer are shown in cartoon presentation, two bound sucrose molecules in each channel in sticks-and-ball representation. Bound Ca^2+^-ions are highlighted by purple spheres. **(B)** Side view of the simulation system Sucsol-36. The periplasmic side is on the lower site. The porin is depicted in cartoon representation (orange), lipid tails as sticks (dark blue), nitrogen and phosphorus atoms as spheres (dark blue), and sucrose molecules in stick-and-ball representation (green). The system was fully solvated (light blue, ions not shown).

The simulation system was prepared by embedding the refined protein model in the lipid bilayer using the INSANE approach (Pluhackova et al., 2013; Wassenaar et al., 2015). The crystal structure of sucrose-specific porin was taken from the Protein Data Bank (PDB entry 1A0T, see Figure 1A) (Forst et al., 1998). The parameters for sucrose were generated using the GLYCAM06 Carbohydrate Builder. The lipid type and the bilayer composition have been proven to be non-essential for both the porin functions and for the pore properties (Parr et al., 1986; Wiese et al., 1994, 1996). Here, a bilayer patch composed of 331 1-palmitoyl-2-oleoyl-sn-glycero-3-phosphocholine (POPC) lipids was constructed in the fluid phase with equal cell lengths in the membrane plane (*L_*x*_* = *L_*y*_*, thickness of approx. 3.76 nm). The protein membrane system was dissolved with TIP3P water (Jorgensen et al., 1983) in a rhombic box (>36,000 water molecules). *Na*^+^ and *Cl*^−^ ions were added at a physiological concentration of 50 mM to the solution, the protein net charge was compensated by ions. All starting structures were initially energy minimized using the steepest descent algorithm. Two setups constructed from the crystal structure without sucrose (system “ScrY-nosuc”) and with 6 bound sucrose molecules (“SrcY-suc”) were simulated for 100 ns each. Subsequently, the system without sucrose after 100 ns was taken as the start configuration for two additional simulations, with 36 (system “Sucsol-36”) and 72 sucrose molecules (“Sucsol-72”) added to the solvent, respectively. These systems were simulated for 0.5 μs each. A snapshot of one simulation system is shown in Figure 1B, it contains in total more than 173,000 atoms.

### 2.2 Simulation Details

Atomistic molecular dynamics simulations were carried out using the open-source software package GROMACS version 4.6.3 with a time step of 2 fs (Berendsen et al., 1995; Hess et al., 2008). The AMBER99SB-ILDN force field (Hornak et al., 2006; Lindorff-Larsen et al., 2010) for proteins was combined with GLYCAM06 parameters (Kirschner et al., 2008; Tessier et al., 2008) for sucrose and the SLIPIDS force field (Jämbeck and Lyubartsev, 2012a,b,c) for lipids. The cutoff for the van der Waals and the (short-range) Coulomb potential was chosen to 1.2 nm. Short-range electrostatic interactions were calculated explicitly, whereas long-range electrostatic interactions were computed using the Particle Mesh Ewald (PME) method (Darden et al., 1993). Temperature coupling was achieved with the Nose-Hoover scheme with a reference temperature of 310 K and a time constant of 1.0 ps (Nosé, 1984; Hoover, 1985). The semiisotropic pressure coupling was controlled using the Berendsen-thermostat algorithm with a time constant of 5.0 ps (Berendsen et al., 1984). The Lincs algorithm was applied to constrain the bond lengths of the hydrogen atoms to a constant value (Hess et al., 1997). Periodic boundary conditions were applied in all three dimensions to avoid boundary effects caused by a finite simulation system.

Analysis was conducted using in-house routines, GROMACS analysis utilities, and the HOLE program (Smart et al., 1997). Molecular visualization was performed using VMD (Humphrey et al., 1996) and PyMOL (DeLano and Bromberg, 2004).

### 2.3 Potential of Mean Force

The Potential of Mean Force (PMF) was computed using umbrella sampling in order to gain insight into the energetic determinants of sucrose transport through ScrY. In umbrella sampling simulations, the sampling of high-energy regions is improved by adding a biasing “umbrella” potential [e.g., Christ et al. (2010)]. For this purpose, Steered Molecular Dynamics (SMD) simulations were initially carried out by pulling a sucrose molecule through one of the three porins in the trimer (Isralewitz et al., 2001). To avoid ambiguities at the channel openings, a structure with a sucrose bound inside the pore was taken as a start configuration. This sucrose molecule was pulled in both directions (along *z*-axis, 50 ns for each direction). From these pulling simulations, 105 starting structures for subsequent umbrella sampling simulations were extracted with a spacing of the sucrose positions along the channel axis of ≤0.08 nm (in total 103 simulations for the mutant). The umbrella potential was introduced between the center of mass of the channel and the sucrose. The harmonic force constant was chosen to 1,000 kJ/mol/nm^2^. Other molecular dynamics parameters were identical to the equilibrium simulations as described above.

**Figure 2:**
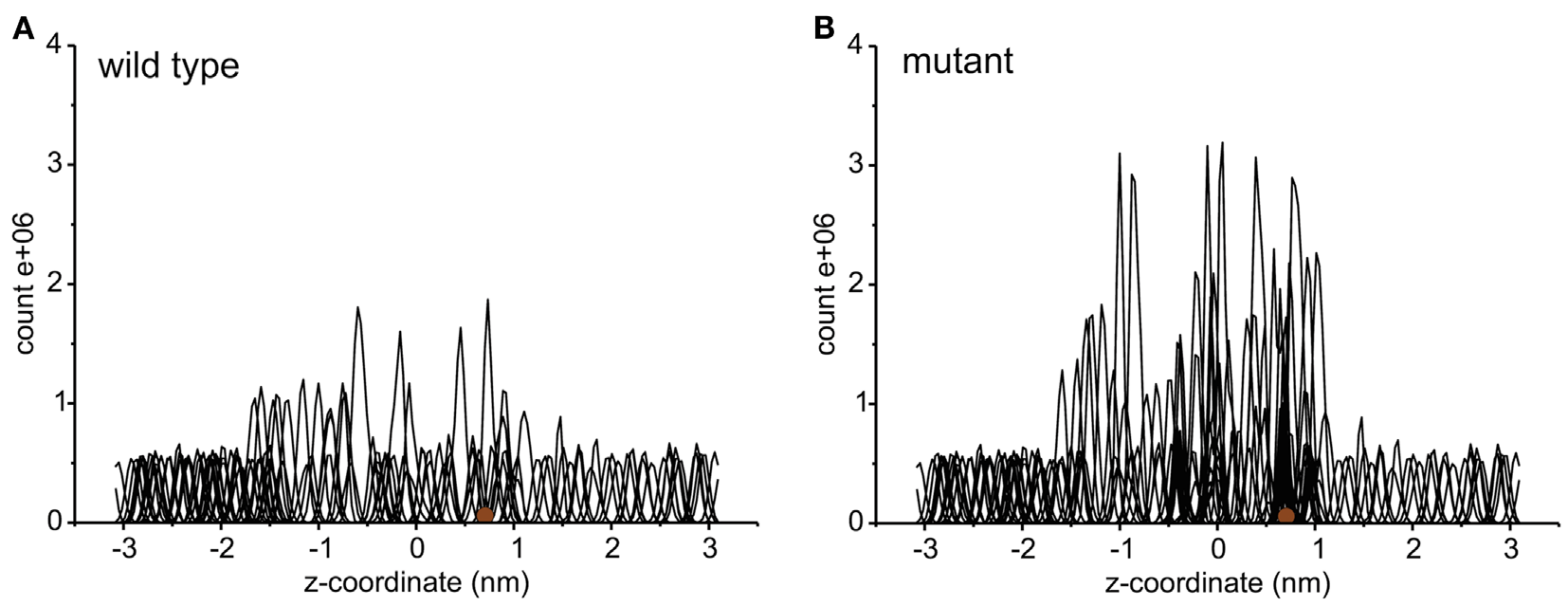
**The histograms of umbrella sampling both for the wild type (A) and for the mutant protein (B)**. The brown sphere depicts the initial position of the sucrose molecule.

Each umbrella was simulated for at least 10 ns. Several samples where the sucrose was close to the pore constriction were equilibrated for longer times in order to obtain an improved equilibration of the system. Histograms distal to the mutation site and the constriction region were used both for the wild type and the mutant. Figure 2 shows the umbrella histograms exhibiting sufficient overlap between the adjacent windows. The potential of mean force as a function of the channel coordinate (*z*-coordinate along the membrane normal) was calculated using the Weighted Histogram Analysis Method (WHAM) based on these umbrella sampling simulations (Rosenbergl, 1992) as implemented in the Gromacs tool g_wham (Hub et al., 2010). The error was obtained from bootstrapping of the histograms using *Bayesian bootstrap* with 200 bootstraps. The PMF was analyzed for a periodic reaction coordinate, and the integrated autocorrelation times of the bootstrapped trajectories were smoothed using a Gaussian filter (Hub et al., 2010).

## 3 Results

The ScrY-trimer with and without bound sucrose were stable in 100 ns simulations with comparably small root mean square deviations (rmsd) of 1–1.5 Å for the backbone atoms of each monomer (not shown, similar rmsd for backbone atoms of trimer structure empty/with bound sucrose). The spontaneous binding of sucrose to the channel was addressed in two 0.5-μs simulations for different sucrose concentrations in solution (36 and 72 sucrose molecules, corresponding to a concentration of ~50 and 100 mM in solution, respectively).

### 3.1 Sucrose Binding to ScrY

**Figure 3:**
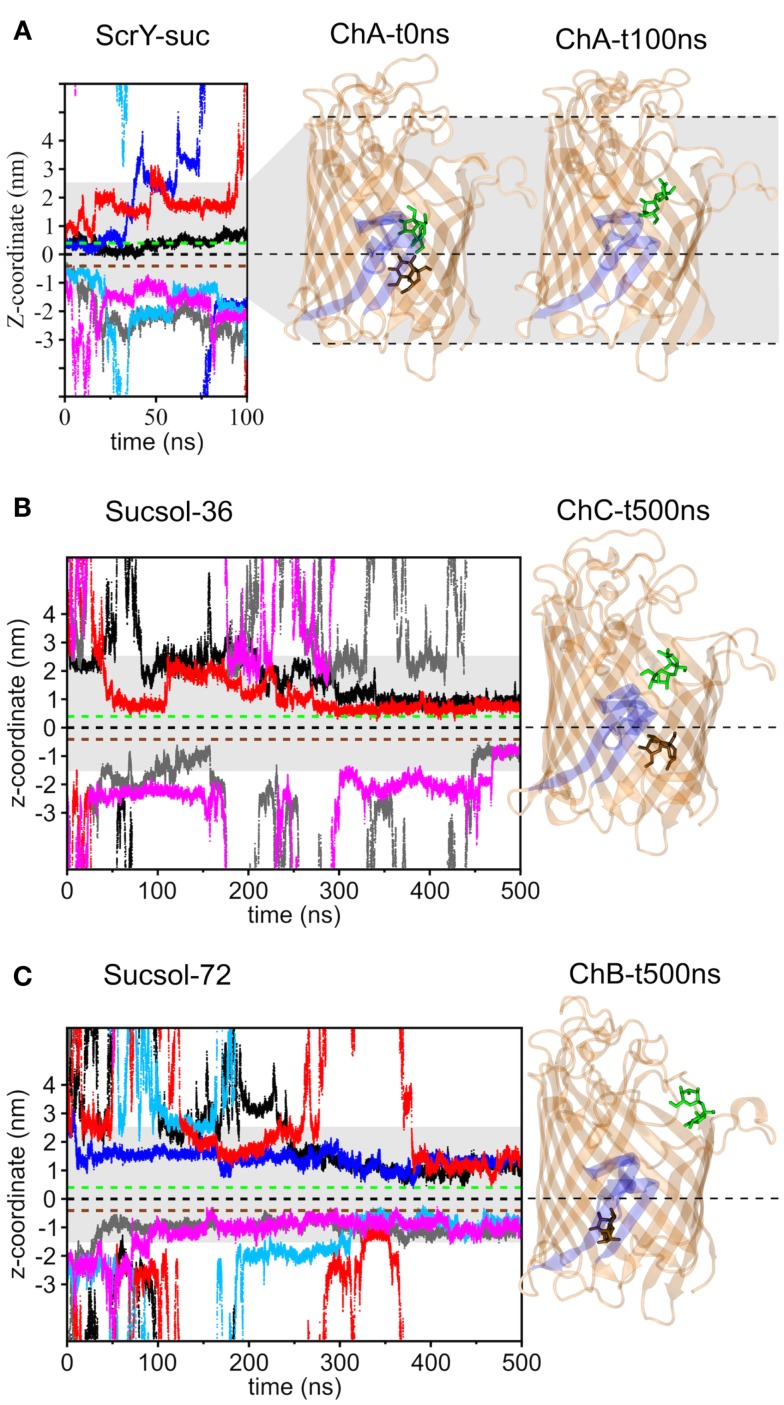
**Displacement of the sucrose molecules along the pore axis (*z*-coordinate) as a function of simulation time for the simulation systems ScrY-suc (A), Sucsol-36 (B), and Sucsol-72 (C)**. The green and brown dashed lines indicate the external and the periplasmic binding sites for sucrose in the crystal structure. In addition, the channel structures of chain A (ChA) at 0 ns (i.e., the crystal structure) and after 100 ns (ScrY-suc system) are shown in the right panel [**(A)**; The three monomers of the homo-trimer were assigned the labels **(A–C)** to distinguish between them.]. The channel structures of chain C (ChC, Sucsol-36 system) and chain B (ChB, Sucsol-72 system) after 0.5 μs are provided in subfigures **(B,C)**, respectively. The inward folded loop L3 is highlighted in blue. The sucrose molecules bound from the external and the periplasmic sides of the channel are shown in stick representation (green and brown, respectively). The coloring of the sucrose traces is as followes: chain A: black and gray, chain B: blue and light blue, chain C: red and magenta.

Two bound sucrose molecules per monomer were resolved in the crystal structure of ScrY (Forst et al., 1998). In the simulations, the sucrose in the periplasmic binding sites spontaneously left ScrY within the first 20 ns of simulation (see Figure 3A, traces colored light blue, gray, and magenta; sucrose colored brown in ScrY structure). For the external binding sites, only one sucrose molecule stayed bound for the full 100 ns (colored green). It is located above the inner loop L3 and below the more flexible loops connecting the *β*-strands of the barrel at the external side. No sucrose molecule passed the center of the channel defined by the midpoint between the bound sucrose molecules of the crystal structure (*z* = 0 nm). These simulation results together with the reported sucrose occupancies of only 0.71 (periplasmic binding site) and 0.80 (external binding site) in the crystal structure (Forst et al., 1998) suggest that not all ScrY sucrose binding sites are occupied under physiological conditions. In addition, sucrose may be trapped in the region between the inner loop L3 and the free loops at the external side of the channel.

Figures 3B,C show the binding of sucrose molecules from the solution to the apo-ScrY as observed in 0.5 μs simulations (Sucsol-36 and Sucsol-72). No sucrose molecule passed the channel in the simulations. However, a number of events is observed for sucrose binding to both periplasmic and external openings of the channel. Each two sucrose molecules got bound to two of the three chains at intermediate concentrations (Sucsol-36), at high concentration two sucrose molecules were bound to each chain. The binding positions were, however, shifted with respect to the positions reported in the crystal structure. Sample snapshots after 0.5 μs of simulation with marked sucrose positions are provided in Figure 3. In conclusion, sucrose is able to spontaneously move into and bind within the channel close to the periplasmic binding site and within the external trapping area, but is not transported on the submicrosecond timescale.

**Figure 4:**
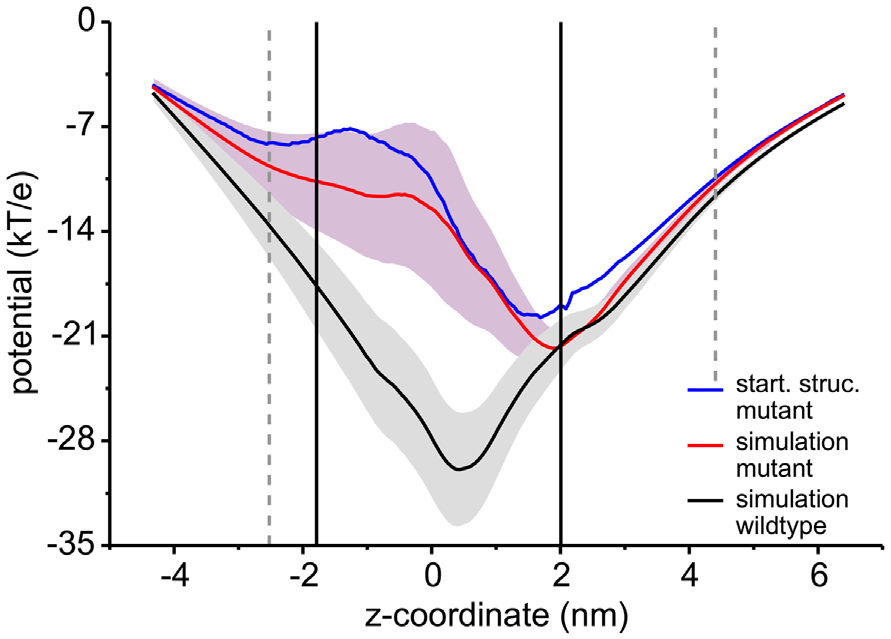
**Electrostatic potential along the channel axis**. The electrostatic potential was calculated using a recently developed Poisson–Boltzmann solver (Bertelshofer et al., 2015). The membrane was implicitly modeled using a dielectric constant of 2 (Böckmann et al., 2008), the region of the membrane boundaries are shown by black lines, the extension of the ScrY protein into the solvent phase by dashed gray lines. The potential for the wild type (black line) was averaged over 100 ns of simulation, the potential for the Asp194Ala:Aps201Ala mutant averaged over 50 ns (red line). The path through the individual channels was analyzed using the HOLE program (Smart et al., 1997). The blue line shows the electrostatic potential for the crystal structure.

In addition to sucrose binding, transport of Na^+^ and Cl^−^ ions could be observed. As detailed in Table 1, in total seven Na^+^ ions and one Cl^−^ ion moved through ScrY during the 100 ns equilibrium simulations (ScrY-nosuc and ScrY-suc), and 33 (1) Na^+^ (Cl^−^) ions during the 0.5 μs simulations (Sucsol-36, Sucsol-72). Thus the passage of positively charged ions through ScrY (both directions) is strongly preferred over the passage of negatively charged ions. This may be explained by the negative electrostatic potential at the external opening and through the pore as shown in Figure 4 that is probably caused by several (negatively charged) aspartic acids in the inner L3 loop of ScrY. Exchange of two aspartic acids of L3 (Asp194 and Asp201) by alanines significantly shifted the electrostatic potential within the pore (see below for the selection of mutants). Interestingly, ion passage was blocked for the wild type ScrY if a sucrose molecule bound close to the inner constriction zone. This is in agreement with earlier experimental studies on porins reporting ion blockage by sugar binding to the porin (Andersen et al., 1998; Kullman et al., 2002).

**Table 1:** **Number of ions passing through ScrY in equilibrium simulations**.

Channel	ScrY-nosuc	ScrY-suc	Sucsol-36	Sucsol-72
Chain A	4 Na^+^	0	9 Na^+^	7 Na^+^
Chain B	0	2 Na^+^	7 Na^+^	4 Na^+^
Chain C	0	1 Na^+^, 1 Cl^−^	4 Na^+^	2 Na^+^, 1 Cl^−^
Sum	4 Na^+^	3 Na^+^, 1 Cl^−^	20 Na^+^	13 Na^+^, 1 Cl^−^

*The three monomers of the homo-trimer were assigned the labels A, B, and C to distinguish between them*.

### 3.2 ScrY Pore Size

**Figure 5:**
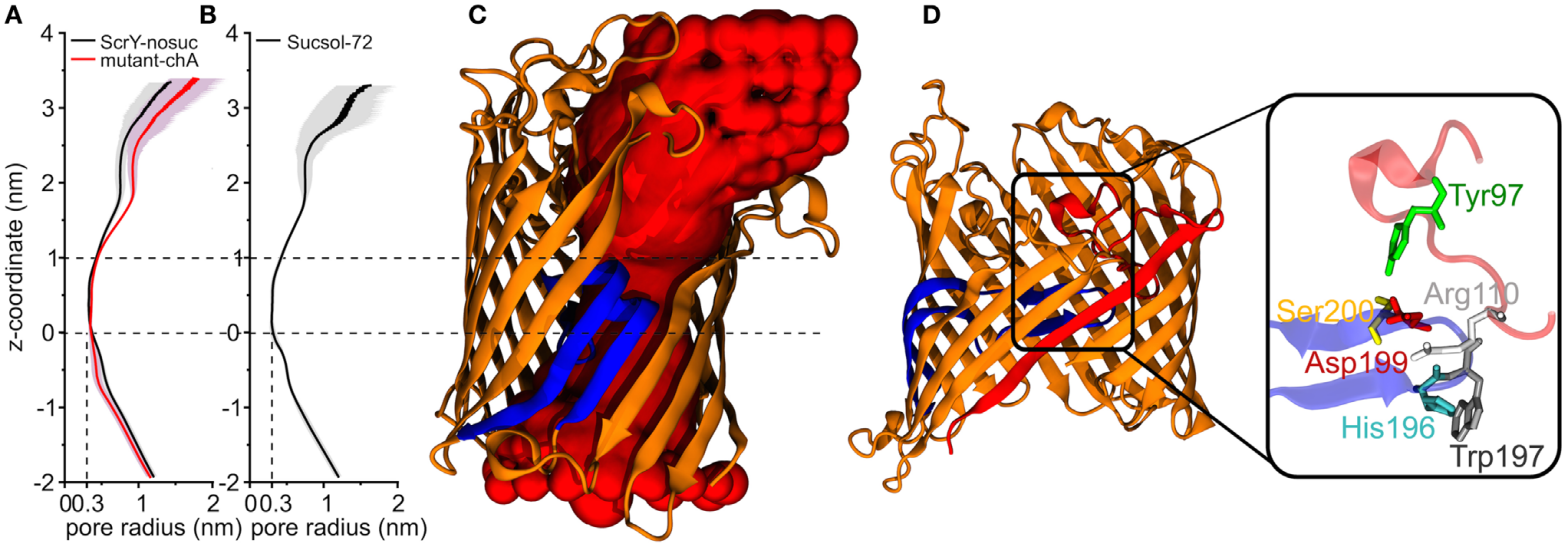
**Pore radius profiles along *z*-coordinate for equilibrium simulations of ScrY without sucrose [ScrY-nosuc, 100 ns, (A)] and mutant [mutant-chA, 50 ns, (A)], and for ScrY in a solution with high sucrose concentration [ScrY-72, 500 ns, (B)]**. The gray shadow describes the flexibility caused by the channel fluctuation. The middle of two crystal sucrose binding sites is found at *z* = 0 nm. The pore for a monomer is visualized **(C)**, where the porin is described by cartoon presentation in orange, and the inner loop L3 in blue. The red spherical probes describe the lumen along the pore axis. The hydrogen bond interactions between loop L1 (red) and loop L3 (blue) of the channel are shown in **(D)**. The detailed description is shown in the enlarged square. The involved amino acids are described by sticks and spheres, where ARG110 (white) from L1 forms hydrogen bonds with HIS196 (blue) and TRP197 (gray) from L3, TYR97 (green) from L1 forms hydrogen bonds with ASP199 (red), and SER200 (yellow) from L3.

The shape and size of the ScrY channel was addressed using the HOLE program (Smart et al., 1996). Figures 5A,B provide both the average pore radius along the channel axis as well the pore flexibility or pore fluctuations (gray shaded area). The radius profile as well as the channel structure (Figure 5C) reflects an hourglass shape of the pore with a minimum radius of only 0.3 nm in the constriction area (0–1 nm). In this region, the pore is narrowed down by the inwardly folded loop L3; it forms the external binding site for sucrose. The loop L3 is stabilized by a hydrogen bond network in particular with loop L1 (Figure 5D). Arg110 of L1 forms hydrogen bonds with His196 and Trp197 (both L3), and Tyr97 (L1) forms hydrogen bonds with Asp199 and Ser200 (both L3).

The external opening of the porin shows a comparable high flexibility. The shape of the pore and also its flexibility appear unchanged for high sucrose concentrations in the medium.

### 3.3 Potential of Mean Force for Passage of Sucrose through ScrY

Umbrella sampling was applied to study the energetics of sucrose passage through ScrY. Starting structures for the individual umbrellas along the channel were extracted from pulling simulations; in these, starting from an initial configuration after 100 ns of simulation (ScrY-suc system, see Figure 3A) a bound sucrose was pulled to both channel openings.

**Figure 6:**
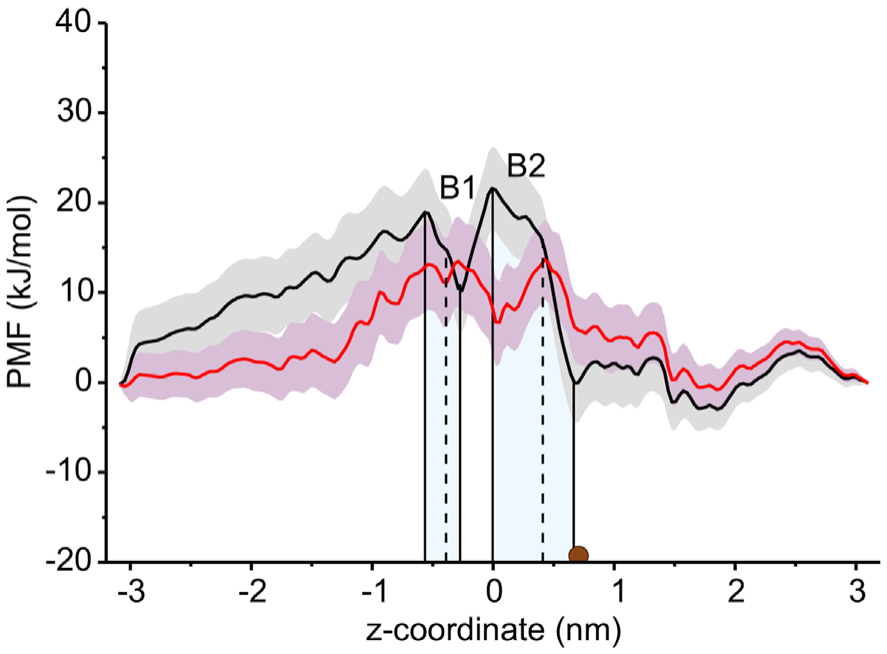
**Potential of mean force (PMF) profiles along the channel coordinate both for the wild type (black) and for the mutant protein (red)**. The shaded areas indicate the statistical uncertainty (67% confidence interval). The two main energy barriers for wt ScrY are highlighted by gray shaded areas (B1, B2). The brown dot depicts the initial position of the sucrose molecule for the initial pulling simulation. The two sucrose binding sites of the crystal structure are found at the dashed lines. The external side of ScrY is found on the right side. The PMF was analyzed using a periodic reaction coordinate in the Weighted Histogram Analysis Method (WHAM), a profile using non-periodic WHAM is provided as Supplementary Information.

**Figure 7:**
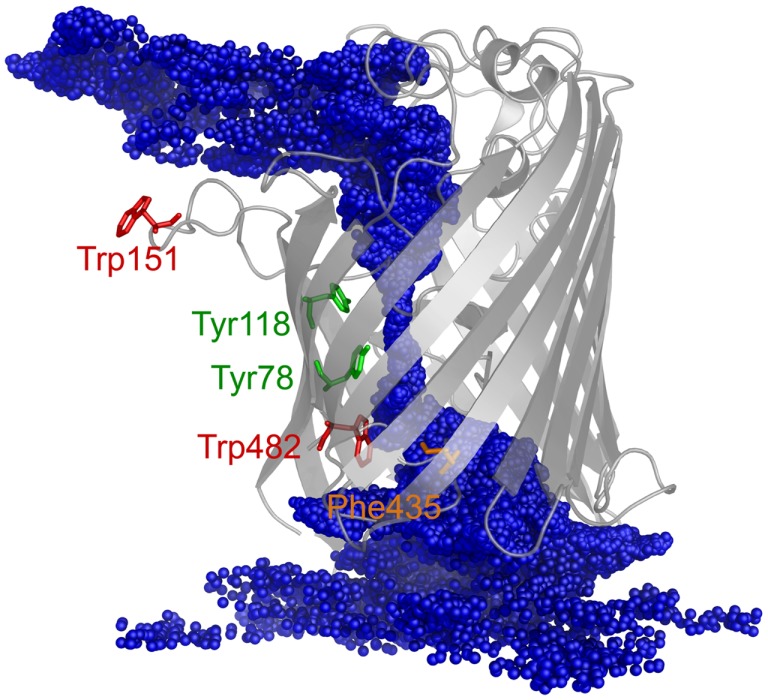
**The ScrY structure is shown together with the center of mass positions for sucrose (blue spheres) sampled during the umbrella sampling simulations (positions recorded every 10 ps)**.

The PMF for sucrose transport through the ScrY using a periodic reaction coordinate shows a total barrier height of ≈ 22 kJ/mol (Figure 6, black line). Two equally steep regions were identified labeled B1 and B2. The first barrier B1 at the periplasmic part of the porin has a height of ≈19 kJ/mol, the second energy barrier B2 of ≈22 kJ/mol. The latter barrier is close to the external sucrose trapping region of ScrY. It is located within the constriction area. The two crystal binding sites for sucrose are found at two metastable positions, right above the B2 barrier, and close to the minimum following the intracellular B1 barrier (dashed lines in Figure 6). Smaller barriers preceding B1 (*z* < −0.6 nm) are caused by electrostatics interactions of sucrose with the mobile loops at the periplasmic pore entrance (not shown). Figure 7 shows sampled sucrose positions from the umbrella simulations. As suggested before, the passage of sucrose mainly follows the amino acids that form the greasy slide (highlighted).

An energy decomposition was applied to determine the influence of all residues aligning the pore through the porin on the passage of sucrose, and key residues were selected for subsequent mutation. The enthalpic contributions of the 115 amino acids forming the ScrY pore on the PMF for sucrose transport were estimated as the sum of the corresponding Lennard-Jones and (short-range) Coulomb interactions (short-range cutoff of 1.2 nm) between each amino acid and the sucrose molecule along the path. The average residue–sucrose interaction energy for each umbrella window was computed and analyzed.

**Figure 8:**
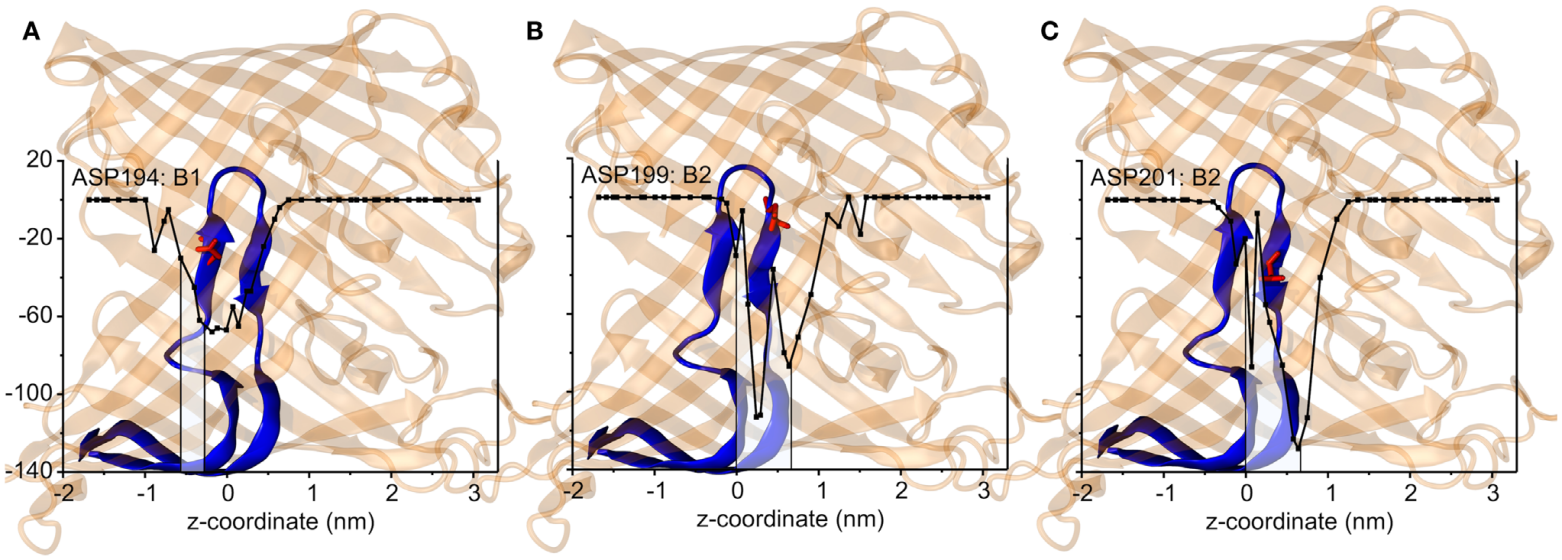
**The short-range Coulomb interactions between the sucrose and three key residues, Asp194 that is responsible for the energy barrier B1 (A), Asp199 for B2 (B), and Asp201 for B2 (C)**. The channel structures are shown by transparent cartoon representation in orange in accordance with the *z* coordinates. The inner loop L3 is shown in blue where the key amino acids are shown as sticks and spheres in red. The middle of two binding sites is presented by z = 0 nm.

**Figure 9:**
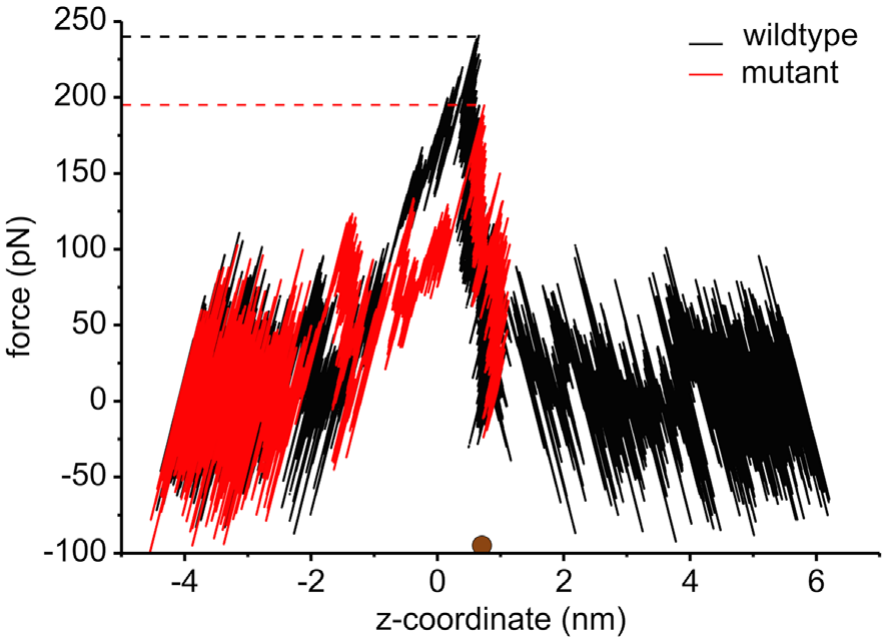
**The pulling force as a function of the displacement of sucrose along the channel coordinate both for the wild type (black) and for the mutant protein (red)**. The brown sphere dot depicts the initial position of the sucrose molecule for the pulling simulation. The force curve for values below ≈0.7 nm is reversed since pulling was performed both to the left and to the right, both pulling simulations starting at the marked position (brown dot). The middle of two binding sites is found at *z* = 0 nm.

The interaction energies of the three most strongly interacting residues with sucrose are shown in Figure 8. Asp194, Asp199, and Asp201 show strong Coulomb interactions, which coincide with the largest barriers of the PMF profile (see Figure 6). Interestingly, these three amino acids are located in the central L3 loop, highlighting the importance of L3 for sucrose transport. For the design of a ScrY mutant with improved sucrose transport characteristics, we accordingly chose Asp194 and Asp201 for mutation to alanine (termed ScrY^mut^). Asp in position 199 was kept due to its role in fixing the position of the L3 loop within the pore (see Figure 5D), as shown above. The influence of the double mutant Asp194Ala:Asp201Ala on the PMF was subsequently tested in additional umbrella simulations, restricted to the region from the external sucrose binding site to the periplasmic ScrY opening. Starting structures for the umbrella simulations were obtained from a sucrose pulling simulation, applied pulling forces for the wt ScrY, and mutant ScrY are compared in Figure 9. The maximal pulling force is reduced from ≈240 pN for wt ScrY to <200 pN for the double mutant. However, considerable fluctuations for the pulling forces are to be expected due to activated processes. Still, the location of the main barrier is in agreement with the PMF result. As expected from the residue-resolved sucrose interaction energies and the pulling forces, the potential of mean force is significantly lowered for the designed ScrY double mutant (Figure 6, red line). Both the B1 and B2 barriers are diminished by the Asp-to-Ala mutations within the L3 loop. The overall barrier height was found decreased by ≈8 kJ/mol. Also the electrostatic potential along the pore axis increased significantly (see Figure 4, green line). Provided the stability of this mutant also *in vivo*, the change in the PMF profile suggests a significantly enhanced sucrose transport for this mutant.

## 4 Discussion

The characteristics of sucrose passage through the porin ScrY was studied using atomistic molecular dynamics simulations. Equilibrium simulations showed that very high sucrose concentrations are required to see (meta-)stable binding of sucrose to both the periplasmic- and the external openings of the porin, i.e., the two binding sites reported for the crystal structure are most probably not simultaneously occupied at low or intermediate sucrose concentrations. The inside width of the hourglass shaped pore and thus the size of molecules allowed for passage is mainly restricted by the inwardly folded L3 loop. It forms a constriction zone with a radius of only 0.3 nm. Larger fluctuations of the pore width were observed only for the external pore mouth, providing a larger access volume for sucrose approach. It has been reported that the sucrose molecule has to form a specific configuration (left-handed helical conformation) in order to pass through this narrow pore (Dutzler et al., 1996; Forst et al., 1998).

The potential of mean force for sucrose passage through the porin was obtained from extensive umbrella sampling simulations. The PMF exhibits two main energy barriers with 19and 22 kJ/mol. The enthalpic contribution to these barriers could be mainly ascribed to Coulombic interactions with the aspartic acids of the central L3 loop (Asp194, Asp199, Asp201), suggesting these acidic residues as mutation candidates for the design of ScrY variants with altered transport characteristics. How well will the porin structure be retained for such mutants? A study of OmpF with four point mutants and one deletion mutant showed no alteration of the barrel structure but only local effects on the structure of the pore constriction region (Lou et al., 1996). The authors mutated pore wall arginines at the constriction zone of OmpF by shorter uncharged residues. Similar to ScrY, OmpF contains acidic residues on the L3 loop and basic residues on the facing barrel wall (Nikaido, 2003). Therefore, one may conclude from the OmpF study that also non-conservative mutations of the counterpart residues of the pore wall arginines, i.e., the mutations of acidic residues of the L3 loop to uncharged ones, will leave the overall porin structure unaltered.

Since one of the aspartic acids (Asp199) is involved in hydrogen bonding with loop L1, that appears essential for the configurational stabilization of loop L3, only two aspartic acids of L3 (Asp194 and Asp201) were mutated to ALA in order to generate a mutant showing enhanced sucrose passage capabilities. Comparisons of forces for pulling sucrose through the porin, of the electrostatic potential along the pore, and of the PMF between wt ScrY and ScrY^mut^ showed a significantly altered energetics for sucrose passage. The PMF barrier is decreased by ≈8 kJ/mol. However, while sucrose passage through ScrY^mut^ will be enhanced, the mutation will possibly also affect the specificity of the porin. Additionally, despite using umbrellas being very close in space with long simulation times (total simulation time for wt was 1.29 μs and for the mutant 1.72 μs), the PMFs are not fully converged and show a substantial error. Still, the results indicate a substantially improved passage of sucrose through the mutated porin.

In summary, key residues for passage of sucrose through ScrY were identified and a double mutant with improved transport characteristics designed *in silico*. The suggested mutant is currently further characterized in experiments.

## Author Contributions

RB designed research, LS performed simulations, LS, FB, and RB performed analysis, and all wrote the manuscript.

## Conflict of Interest Statement

The authors declare that the research was conducted in the absence of any commercial or financial relationships that could be constructed as a potential conflict of interest.
