# Supplementary material for: Characteristics of Sucrose Transport through the Sucrose-Specific Porin ScrY Studied by Molecular Dynamics Simulations
Source: Front Bioeng Biotechnol. 2016 Feb 15;4:9. doi: 10.3389/fbioe.2016.00009 (PMC4753733; doi:10.3389/fbioe.2016.00009)
Supplement: Supplementary file 1 [file data_sheet_S1.PDF]

Supplementary Information: Characteristics of Sucrose Transport through the Sucrose-Specific Porin ScrY Studied by Molecular Dynamics Simulations

Liping Sun [1], Franziska Bertelshofer [1,2], Günther Greiner [2], Rainer A. Böckmann [1,*]

[1] *Computational Biology, Department of Biology, Friedrich-Alexander University of Erlangen-Nürnberg, Erlangen, Germany*
[2] *, Computer Graphics Group, Department of Computer Science, Friedrich-Alexander University of Erlangen-Nürnberg, Erlangen, Germany*

Correspondence*:
Rainer A. Böckmann
Computational Biology, Department of Biology, Friedrich-Alexander University of Erlangen-Nürnberg, Staudtstrasse 5, Erlangen, 91058, Germany,
rainer.boeckmann@fau.de

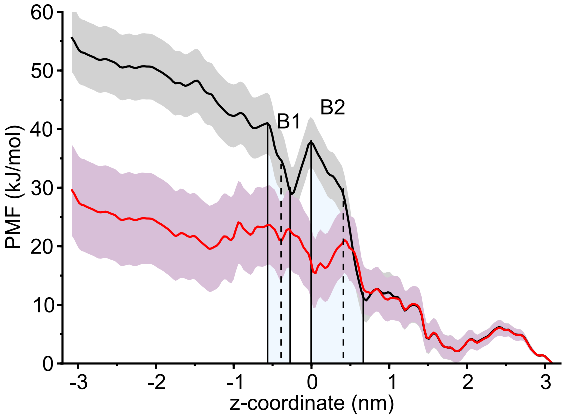

Figure 1. Potential of mean force (PMF) profiles along the channel coordinate both for the wild type (black) and for the mutant protein (red), using non-periodic Weighted Histogram Analysis Method (WHAM). The shaded areas indicate the statistical uncertainty (67% confidence interval). The two main energy barriers for wt ScrY are highlighted by grey shaded areas (B1, B2). The brown dot depicts the initial position of the sucrose molecule for the initial pulling simulation. The two sucrose binding sites of the crystal structure are found at the dashed lines. The external side of ScrY is found on the right side.
